# Simultaneous Determination of Acrylamide and Hydroxymethylfurfural in Extruded Products by LC-MS/MS Method

**DOI:** 10.3390/molecules24101971

**Published:** 2019-05-22

**Authors:** Antun Jozinović, Bojan Šarkanj, Đurđica Ačkar, Jelena Panak Balentić, Domagoj Šubarić, Tanja Cvetković, Jasmina Ranilović, Sunčica Guberac, Jurislav Babić

**Affiliations:** 1 Faculty of Food Technology Osijek, Josip Juraj Strossmayer University of Osijek, Franje Kuhača 20, 31000 Osijek, Croatia; ajozinovic@ptfos.hr (A.J.); jelena.panak@ptfos.hr (J.P.B.); jbabic@ptfos.hr (J.B.); 2 Department of Food Technology, University Centre Koprivnica, University North, Trg dr. Žarka Dolinara 1, 48000 Koprivnica, Croatia; bojan.sarkanj@unin.hr; 3 Faculty of Chemical Engineering and Technology, University of Zagreb, Marulićev trg 19, 10000 Zagreb, Croatia; dsubaric@fkit.hr; 4 Research and Development, Podravka d.d., Ante Starčevića 32, 48000 Koprivnica, Croatia; tanja.cvetkovic@podravka.hr (T.C.); jasmina.ranilovic@podravka.hr (J.R.); 5 Faculty of Agrobiotechnical Sciences Osijek, Josip Juraj Strossmayer University of Osijek, Vladimira Preloga 1, Osijek 31000, Croatia; suncica.guberac@fazos.hr

**Keywords:** acrylamide, hydroxymethylfurfural, LC-MS/MS method, extruded snack products

## Abstract

The aim of this study was to develop a liquid chromatography/tandem mass spectrometry LC-MS/MS method for the simultaneous determination of acrylamide and hydroxymethylfurfural (HMF) in corn snack products enriched with food industry by-products: brewer’s spent grain (BSG), sugar beet pulp (SBP) and apple pomace (AP). Development of the method included the study of different sources for ionization, different mobile phases, different extraction conditions as well as different methods of sample preparation. Finally, the single LC-MS/MS method was developed for the analysis of both analytes in one step with a duration of 20 min using a simple single-step extraction. The method with apparent recoveries of 91.4 and 90.4 for acrylamide and HMF, respectively, was applied for the analysis of non-extruded and extruded samples. The obtained results shown that the acrylamide content was <LOD (limit of detection) for all raw materials and non-extruded mixtures, while HMF increased proportionally to the content of added by-products in the mixtures. After the extrusion process, quantification of the acrylamide could be done in all samples. A higher amount of by-products entails higher contents of acrylamide and HMF, with the most significant effect in AP extrudates, where the highest content of HMF (6069 ± 789 ng/g) and acrylamide (5.37 ± 0.50 ng/g) in samples with 15% AP was observed.

## 1. Introduction

Acrylamide and hydroxymethylfurfural (HMF) have received great attention in scientific papers and from the European Food Safety Authority (EFSA) in the last decade, because of their wide occurrence in diverse foods and their toxic potential. The primary mechanism of formation of those contaminants in food is associated with the Maillard reaction during the processing of food rich in carbohydrates and proteins at high temperatures [1,2,3,4,5]. HMF is one of the most important intermediates of well-known Maillard reactions and is an early marker of these reactions, since it is formed in the first steps, and occurs in many foods rich in carbohydrates [6]. In addition, this cyclic aldehyde is also formed during the acid hydrolysis of hexoses by eliminating three molecules of water [7]. Thus, it is important to emphasise that HMF is not only generated by Maillard reactions, but also during the degradation of hexose and caramelization, which does not require the presence of an amino group [8]. In addition to temperature as the main factor, the rate of HMF formation in food also depends on the type of sugar, pH, water activity and the proportion of divalent cations [3]. It is especially present in over-processed foods, and its impact on health is still a subject of numerous discussions. Special concern for HMF is related to its conversion to 5-sulphoxymethylfurfural (SMF) and 5-chloromethylfurfural (CMF), for which cytotoxic, nephrotoxic, mutagenic and carcinogenic properties have been established, as well as the emergence of colon, liver and skin cancer [3,6,9]. The presence of HMF is characteristic in bakery products and biscuits, and can be also found in honey, fruit and vegetable products (pastas, juices), baby food, beer, etc. [3,4,10,11,12]. Although there are no maximum values prescribed for snack products, the Codex Alimentarius and the European Union [13,14] prescribed a maximum amount of HMF in honey (40 mg/kg) and apple juice (50 mg/kg).

In the literature there are different methods for the determination of HMF, which can be classified in three main groups: colorimetric, spectrophotometric and chromatographic methods [15]. High performance liquid chromatography (HPLC) with a UV detector (about 280 nm wavelength) is the most commonly used method for the determination of HMF in numerous food products [2,10,11]. However, the main disadvantage of this method is that many other compounds naturally present or formed in food during processing may also absorb these wavelengths, which may negatively affect the quantification of HMF by UV detection [8,9]. Therefore, more selective methods based on mass spectrometry (MS) have been developed recently. liquid chromatography/mass spectrometry (LC-MS) and liquid chromatography/tandem mass spectrometry (LC-MS/MS) methods are used for the determination of HMF in different kinds of products, usually performed by the selected-ion monitoring (SIM) mode or multiple reaction monitoring (MRM) mode, with *m*/*z* 109 and /*m*/*z* 127 as the monitored ions [5,8,9].

Acrylamide is one of the Maillard products which has genotoxic and carcinogenic properties, and in high doses has neurotoxic properties, according to the International Cancer Research Agency (IARC). Because of that, it is classified as probably carcinogenic to humans (group 2A) by the IARC [16]. The presence of acrylamide in food products has raised major concern in the early 2000s, mainly by the Swedish National Food Agency and researchers from University of Stockholm [17,18]. Since then, a large amount of research has been performed with the aim to determine the causes of the presence of acrylamide in food products, as well as the possible reaction pathways of its formation. It has been concluded that the major reaction pathway of the acrylamide formation is the Maillard reaction, in which the naturally present amino acid (asparagine) reacts with reducing sugars when the environment is exposed to heat [19,20,21,22]. Although acrylamide in foods is primarily formed during the Maillard reaction, there are other less significant pathways of formation. One of them is referred to as acrolein and acrylic acid, which may be formed by the dehydration of glycerol, especially if the fat is heated at improperly high temperatures. In addition, acrylamide can be formed together with ammonia during the degradation of amino acids, as well as from wheat gluten, where amino acid alanine is a key precursor [3,23]. Considering the potential adverse effects of acrylamide on the human organism, numerous studies have focused on its reduction in food. It was found that the moisture content has a significant influence on its formation during processing and typically higher values occur at lower moistures. Furthermore, the chemical composition of raw materials is also significant, where the content of asparagine and reducing sugars is the most important parameter [24]. The use of asparaginase has been described as an effective mitigation strategy to reduce acrylamide both in potato-based products [25], and in cereal-based products, where acrylamide reduction was found to be up to 90% without affecting the organoleptic properties of the products [2,26]. Furthermore, since 2002, the European Commission has made considerable efforts to investigate the way in which acrylamide is formed and to reduce its levels in processed foods. Thus, in 2007, the European Commission issued the first recommendation on the monitoring of acrylamide levels in food [27]. Similar recommendations were issued in 2010 [28] and 2013 [29], while in 2017 the Commission Regulation 2017/2158 [30] established mitigation measures and benchmark levels for the reduction of the presence of acrylamide in food. Although this regulation prescribes benchmark levels for the presence of acrylamide in different foodstuffs, directly expanded corn snack products are not included in the legislation. However, the values that could be referential ones are those prescribed for breakfast cereals based on corn (150 μg/kg) [30]. In addition, in 2012, the EFSA collected monitoring results from 2007 to 2010 and published them in a scientific report [31], and in 2015 the Scientific Panel on Contaminants in the Food Chain (CONTAM) of the EFSA adopted an opinion on acrylamides in food [32].

The quantification of acrylamide in foods is a challenge due to its low molecular weight (71.08 Da), high polarity, very good solubility in water (215.5 g/100 mL), high reactivity and low volatility [33,34]. Furthermore, the main problem for quantification in complex systems, such as food, are compounds that interfere with detection due to the matrix effect, and their losses during extraction and sample preparation [34]. Unfortunately, there are still no standardized methods for the determination of acrylamide in foods. Current methods are severely inconvenient, expensive and time consuming. Several methods have been shown to be sufficient in acrylamide detection, such as gas chromatography-mass spectrometry (GC-MS) and liquid chromatography with tandem mass spectrometry (LC-MS/MS). These methods are currently mostly used for acrylamide detection, while many others have been developed [35]. For the purpose of validation, recovery correction and matrix effect reduction during the analysis, regardless of the type of method, the internal standard ^13^C_3_-acrylamide is used [36]. In so far published analyses performed by LC-MS and LC-MS/MS, usually the ions with *m*/*z* 72 and *m*/*z* 55 for acrylamide, or *m*/*z* 75 and *m*/*z* 58 for ^13^C_3_-acrylamide were monitored. The most popular ionization techniques used in these methods are electrospray ionization (ESI) and chemical ionization under atmospheric pressure (APCI), which are considered to be “gentle” ionization techniques that enable the detection of very polar substances such as acrylamide [34].

The formation of acrylamide and HMF during the extrusion process is not sufficiently investigated, despite the fact that the production of directly expanded snack products includes the application of high temperatures, which could cause the formation of these unwanted compounds. Furthermore, extruded snacks are consumed in high quantities among all generations, from babies to elderly people [37], representing an important reason to investigate the content of acrylamide and HMF in these products. To the extent of our knowledge, all so far published methods for the determination of acrylamide and HMF were developed for separate determination. Therefore, the objectives of this study were: (a) to develop a LC-MS/MS method for the simultaneous determination of acrylamide and hydroxymethylfurfural (HMF); (b) identification and quantification of acrylamide and HMF in corn snack products enriched with food industry by-products; (c) evaluation of HMF and acrylamide content in relation to non-extruded samples and determination of the influence of the process and raw-materials.

## 2. Results and Discussion

**Figure 1:**
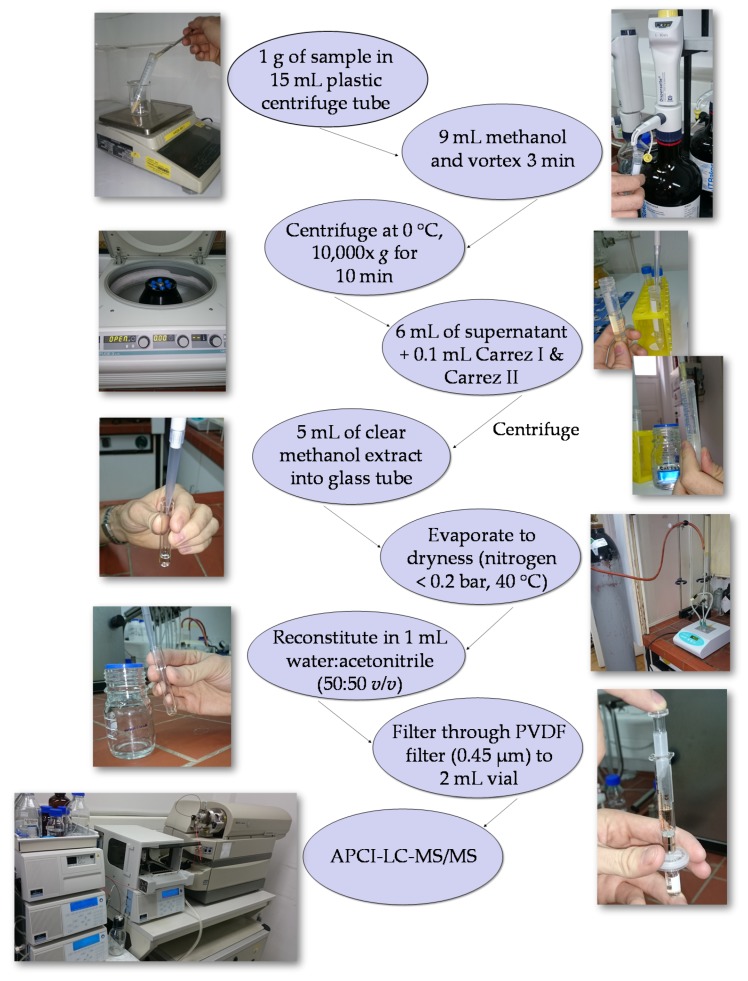
The liquid chromatography/tandem mass spectrometry (LC-MS/MS) method developed for the determination of acrylamide and hydroxymethylfurfural (HMF) in extruded and non-extruded corn-based samples.

With the aim to develop a new LC-MS/MS method for the simultaneous determination of both compounds in one step, in this study different conditions were tested. So, the development of the method included the comparison of different sources for ionization (ESI—electrospray ionization; APCI—chemical ionization under atmospheric pressure), the usage of different types of gradients and mobile phases (water acidified with formic and acetic acid, and acetonitrile), the use of different types of extraction (aqueous and methanolic) and methods of sample preparation (single and multiple extraction, with or without concentration, and with or without purification with the SPE (Solid Phase Extraction) columns). Finally, the single LC-MS/MS method was developed for analysis of both analytes within 20 min using simple single step extraction. The obtained method is described in the section Materials and Methods and is illustrated in Figure 1.

The analytical performance of the method was validated and all validation parameters were summarized in the Table 1. According to the obtained results, it is evident that the signal responses could be accepted to be linearly related to the injected concentration ranges for all analytes, because the regression coefficients (R^2^) of the straight-line graphs were above 0.99. Limit of detection (LOD) and limit of quantification (LOQ) values were 0.62 and 1.89 ng/g for acrylamide, 0.58 and 1.75 ng/g for ^13^C_3_-acrylamide and 18.9 and 57.5 ng/g for HMF, respectively. Furthermore, Table 1 shows that acrylamide and ^13^C_3_-acrylamide eluted at 9.10 min, while HMF was eluted at 13.3 min, all with satisfactory retention time reproducibility.

**Figure 2:**
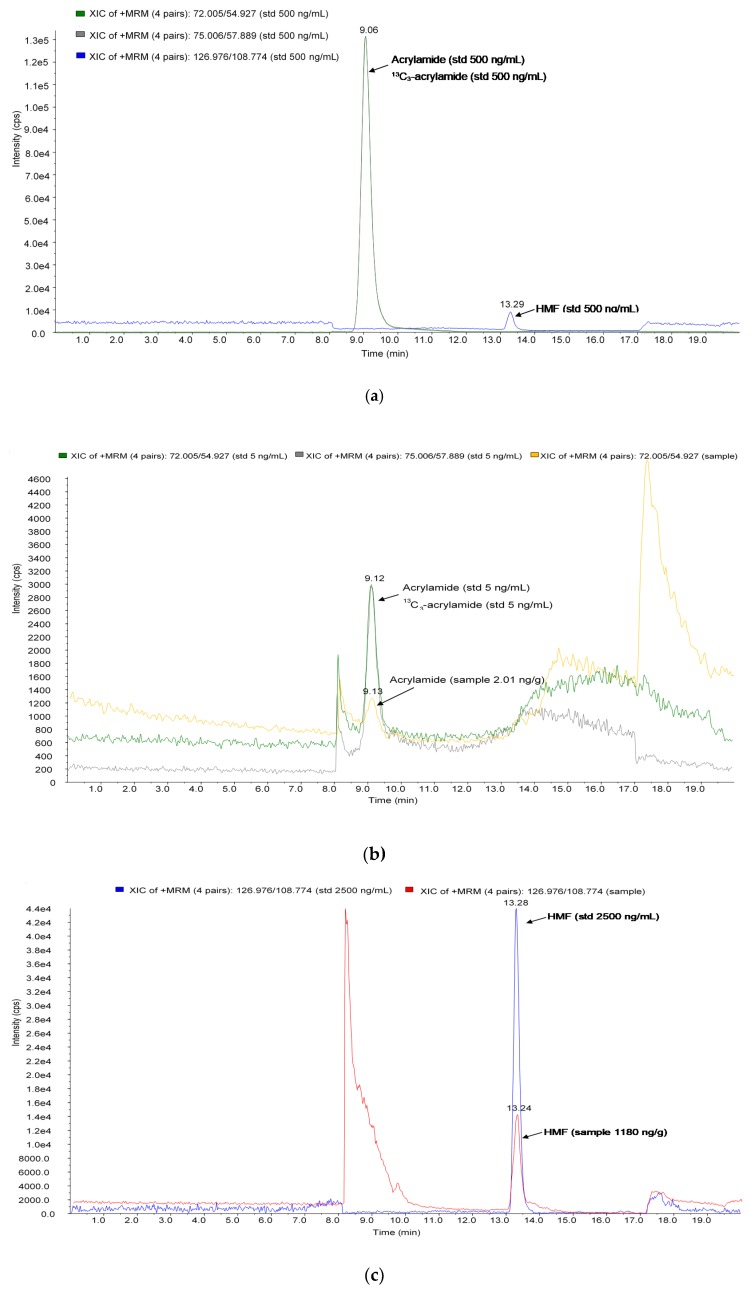
Chromatograms of the: (**a**) standard solution of acrylamide, ^13^C_3_-acrylamide and HMF (500 ng/mL); (**b**) standard solution of acrylamide and ^13^C_3_-acrylamide (5 ng/mL) and extruded sample with detected 2.01 ng/g of acrylamide; (**c**) standard solution of HMF (2500 ng/mL) and extruded sample with detected 1180 ng/g of HMF.

Results obtained during the analysis of spiked samples show that the mean percentage of apparent recovery (R_A_), extraction efficiency (RE) and matrix effect (SSE) exceeded 90% for all analytes (Table 1). Furthermore, the low values of relative standard deviation (RSD) for intra- and inter-day precision were obtained. Therefore, it could be concluded that the method is suitable for the detection of relevant concentrations of acrylamide and HMF. In this study, ^13^C_3_-acrylamide as the internal standard (IS) was used at the beginning as a precautionary step in case of a high matrix effect or low recoveries. All of the relevant parameters were within satisfactory limits, and ^13^C_3_-acrylamide as the IS was left out of further analyses, but remained in the standards which we prepared as controls for retention time, and batch-to-batch signal controls. Due to high price of ^13^C_3_-acrylamide, it was not used for corrections of recoveries and matrix effects within measured matrices, but in cases of different matrices, it can be quickly added and used if needed. Figure 2 shows the chromatograms obtained for the standard solution and extruded sample.

The method developed in this research was used for the analysis of non-extruded mixtures and extruded corn snack products enriched with three different food industry by-products: brewer’s spent grain (BSG), sugar beet pulp (SBP) and apple pomace (AP). Since the objective of our previous research [38] was to produce corn snack products with acceptable physical and sensory properties, it was found that in the case of BSG and SBP 1% d.m. of pectin must be added in the mixtures prior to extrusion.

The results of the acrylamide and HMF analyses of the raw materials are shown in Table 2. It is evident that the acrylamide values were under the LOD (<0.62 ng/g) in all raw materials, while the HMF content ranged from 63.0 ± 3.00 ng/g in the corn grits to 16,019 ± 978 ng/g for the AP.

The effect of the extrusion procedure and by-product addition on the acrylamide and HMF content is shown in Table 3. In accordance with the results obtained for the raw materials, the acrylamide content was under the LOD in all non-extruded mixtures. After the extrusion process, acrylamide could be detected and quantified in all samples. A higher amount of by-products resulted in higher acrylamide contents, regardless of the used by-product. Thus, the highest contents of acrylamide were recorded in samples with 15% added BSG and SBP (3.13 ± 0.23 and 3.00 ± 0.21 ng/g), and significantly the greatest in the sample with 15% AP (5.37 ± 0.50 ng/g), which was more than twice of the content recorded in the extruded sample of corn grits (2.25 ± 0.29 ng/g).

The content of HMF increased proportionally to the content of by-products in non-extruded mixtures. The most significant increase was observed in the application of AP, so the sample with 15% AP had the highest content of HMF (1277± 11.0 ng/g). The extrusion process caused an increase in HMF content for all samples. The extruded corn grits had the lowest value of HMF (174 ± 2.21 ng/g), while in the case of samples with by-products, higher values were recorded in samples with 15% added BSG and SBP (301 ± 12.6 and 316 ± 9.47 ng/g) and was significantly the highest in the sample with 15% AP (6069 ± 789 ng/g).

The significantly higher contents of acrylamide and HMF in samples with AP, compared to samples with BSG and SBP, lies in the mechanism of formation of these compounds during food processing, which is related to the Maillard reaction between reducing sugars and amino acids. As AP contains a significantly higher content of these sugars, the obtained results are consistent with this fact. Confirming this are the results of the carbohydrate contents of these by-products, published in our previous article [38]. So, the content in AP was 95.9% d.m., while in BSG and SBP it was significantly lower (59.9% and 84.3% d.m.).

Furthermore, the significantly lower values of acrylamide content compared to HMF could be attributed to the lack of free asparagine in all samples, so the asparagine portion could be listed here as a limiting factor for acrylamide formation in a larger proportion. This is confirmed by research conducted by Masatcioglu et al. [26], who did not record acrylamide i.e., it was <LOD in the control sample of corn grits, due to the lack of asparagine and reducing sugar in corn grits.

So far research related to the analysis of acrylamide and HMF has mainly focused on different groups of bakery products (bread, biscuits, crackers, etc.), fried products (chips, french fries etc.) and breakfast cereals. From the above product groups, breakfast cereals are the most similar to corn snack products. Accordingly, Teixidó et al. found that HMF content in breakfast cereals ranged from 24.7 to 46.8 μg/g in the first study using the GC-MS method [39], and 27.2–47.2 μg/g by the LC-MS/MS method in another study [8]. Similar results were obtained in research performed on numerous foodstuffs, where the HMF content in breakfast cereals ranged from 12.6 to 46.2 mg/kg [6]. Obviously, all values of HMF content found in breakfast cereals were significantly higher than the values obtained for expanded snacks in this study. Results for HMF obtained in this study are more similar to the results of the research conducted by Gökmen and Şenyuva on 16 commercial baby food samples (three milk-based and 13 cereal-based) [9], who found that all samples based on milk, as well as eight cereal-based samples had <1 μg/g of HMF, while other cereal-based samples had up to 5 μg/g of HMF, with the exception of one sample which had 57.2 μg/g. Accordingly, the highest value of HMF observed in the extruded sample with 15% AP (6069 ± 789 ng/g) remains in the range obtained in the aforementioned study.

Previous investigations of acrylamide content are primarily related to various bakery and fried products (bread crisp, biscuits, french fries, potato chips, etc.). However, it is important to highlight previous published studies on the formation of acrylamide in corn extrudates [26] as well as in potato flour and semolina extrudates [24]. Thus, Mulla et al. [24] concluded that increasing the proportion of potato flour in the mixture affects the increase of acrylamide formation. So, the highest value for acrylamide was recorded in the extruded mixture of potato flour:semolina (70:30) (607 ± 1.00 μg/kg) and the lowest in the mixture (30:70) (221 ± 14.0 μg/kg). In addition, the moisture content and temperature during extrusion had a greater impact on the formation of acrylamide in relation to screw speed. Masatcioglu et al. [26] investigated the effects of different extrusion processes (classical and extrusion with CO_2_ injection), reducing sugars (D-glucose and D-ribose), moisture content and die temperature, as well as chemical leavening agents (sodium bicarbonate –NaHCO_3_ and ammonium bicarbonate –NH_4_HCO_3_), and citric acid on acrylamide formation in corn-based extrudates. They stated that the increase in moisture content and application of CO_2_ extrusion significantly influenced the reduction of acrylamide formation. Furthermore, the sugar type had no significant effect on acrylamide formation, whereas the addition of chemical leavening agents contributed to an increase in acrylamide content, with a more significant influence from NH_4_HCO_3_. The results obtained in both aforementioned studies on acrylamide content were significantly higher than those obtained in this research. An explanation for this was given by Masatcioglu et al. [26] in the introduction of their paper, where they noted that the extrusion process due to its short duration should result in low acrylamide content. For this reason, with the aim of obtaining a higher content of acrylamide these researchers “initiated” its formation during extrusion with the addition of reducing sugars (d-glucose and d-ribose) and asparagine in the mixture. On the other hand, Mulla et al. [24] recorded significantly higher acrylamide content in their extrudates due to the significant content of free asparagine found primarily in potato (2010–4250 mg/kg) and slightly lower in wheat (167–270 mg/kg).

Finally, it is certainly important to note that the obtained values for acrylamide and HMF in this study are considerably lower than the benchmark levels prescribed by the European Commission for the acrylamide content in different foodstuffs [30], and the Codex Alimentarius and the European Union for the content of HMF in honey and apple juice [13,14]. These recommendations do not directly relate to a group of corn snacks products, as these products do not currently have the maximum permissible content of acrylamide and HMF. The obtained results in this study indicate that the extrusion process can be used for the production of directly expanded corn snack products that are safe for consumers with low contents of the above-mentioned potentially harmful compounds for humans.

## 3. Materials and Methods

### 3.1. Chemicals and Consumables

High purity acrylamide, ^13^C_3_-acrylamide and 5-hydroxymethylfurfural were purchased from Sigma-Aldrich (Diesenhofen, Germany). HPLC gradient grade acetonitrile and methanol were purchased from J.T. Baker (Deventer, Holland). All other chemicals used in this study were of analytical grade. Zorbax Extend-C18 column (250 × 4.6 mm, 5 μm) was purchased from Agilent Technologies (Santa Clara, CA, USA), and 0.45 μm PVDF (polyvinyl difluoride) filters Chromafil Xtra PVDF-45/25 from Macherey-Nagel (Düren, Germany). Ultra-pure water was used throughout the experiments (MilliQ system, Millipore, Bedford, MA, USA).

Stock solutions of acrylamide (1 mg/mL), ^13^C_3_-acrylamide (1 mg/mL) and HMF (1 mg/mL) were prepared by dissolving in distilled water:acetonitrile (50:50 *v*/*v*). Working solutions were prepared by diluting the stock solution with water:acetonitrile (50:50 *v*/*v*) to concentrations of 0.75, 1, 2.5, 5, 10, and 25 ng/mL for acrylamide and ^13^C_3_-acrylamide; 50, 100, 250, 500, 1000 and 2500 ng/mL for HMF. Stock solutions and working standards were kept at 4 °C. Carrez I solution was prepared by dissolving 15 g of potassium hexacyanoferrate in 100 mL of water, and Carrez II solution by dissolving 30 g of zinc sulfate in 100 mL of water.

### 3.2. Preparation of Corn Snack Products

Raw materials for the production of corn snack products (corn grits, brewer’s spent grain (BSG), sugar beet pulp (SBP), apple pomace (AP) and pectin), as well as the blend preparation for the extrusion process were described in our previous article [38]. For the purpose of analysis of acrylamide and HMF, samples with 5%, 10% and 15% d.m. of added by-products were produced, whereby the samples with BSG and SBP were prepared with addition of 1% d.m. of pectin.

Extrusion was performed in a laboratory single-screw extruder (Brabender GmbH, Model 19/20DN, Duisburg, Germany) with the process parameters described previously [38]. The obtained samples were air-dried overnight at room temperature, milled in a laboratory mill (IKA MF10, Staufen, Germany) with a 1 mm sieve and stored in sealed plastic bags at 4 °C until further analysis.

### 3.3. Acrylamide and Hydroxymethylfurfural (HMF) Determination

#### 3.3.1. Sample Preparation

The extraction procedure was performed according to the method of Gökmen et al. [40], with some modifications. The 1 g of ground sample was weighted in 15 mL plastic corning centrifuge tube with cap. The extraction was performed with 9 mL of methanol for 3 min in a vortex mixer. The mixture was cold centrifuged (0 °C) at 10,000× *g* for 10 min. After centrifugation, the 6 mL of clear supernatant was transferred into 15 mL plastic tube, and mixed with 0.1 mL of Carrez I and 0.1 mL of Carrez II reagents for 30 s in a vortex mixer. With the aim to precipitate proteins and other co-extracted colloids, the mixture was left to stand for 20 min at room temperature, and then cold centrifuged (0 °C) at 10,000× *g* for 10 min. After centrifugation, the 5 mL of clear supernatant was transferred into an 8 mL glass tube and evaporated to dryness at 40 °C under a gentle stream of nitrogen (<0.2 bar). The remaining residue was immediately reconstituted in 1 mL of water:acetonitrile (50:50 *v*/*v*) by mixing in a vortex mixer for 2 min. Finally, sample was filtered through 0.45 µm PVDF filter, transferred to 2 mL autosampler vial and 20 µL was injected into LC-MS/MS. All sample extractions and measurements were performed in duplicate.

#### 3.3.2. LC–MS/MS Analysis

LC-MS/MS analysis was performed based on the methods developed by Gökmen i Şenyuva [9,41], with some modifications. A HPLC system (PerkinElmer, Walthman, MA, USA) consisting of a binary pump, an autosampler and temperature controlled column oven, coupled to an API 2000 MS/MS (Sciex, Framingham, MA, USA) detector was used for analysis. The ionization was performed with an APCI (atmospheric pressure chemical ionization) interface and the separation of ions with triple quadropol. The analytical separation was performed on a Zorbax Extend-C18 column (250 × 4.6 mm, 5 μm) with two mobile phases: acetonitrile (mobile phase A) and 0.01 mM acetic acid in 0.2% aqueous solution of formic acid (mobile phase B). Elution was carried out at 25 °C with a flow of 0.3 mL/min, by using the following gradients: at the beginning of the analysis (10% A and 90% B); up to 10 min (increase of mobile phase A to 90% and decrease of mobile phase B to 10%); from 10 to 15 min (retention of achieved conditions: 90% A and 10% B); from 15 min to the end of the analysis (re-equilibration at initial conditions: 10% A and 90% B for 5 min). The total runtime of the analysis was 20 min. With the aim to reduce contamination of the APCI source the first 8 min eluate went to the waste, after which, up to 17 min, eluate was directed to the MS and then again went to waste until the end of the analysis. Ionization was performed in APCI positive mode with the following parameters: curtain gas (20 psi); collision gas (4 psi); ionspray voltage (5000 V); vaporizer temperature (425 °C); sheath gas (50 psi); drying gas (30 psi). The quantification was performed in multiple reaction monitoring (MRM) mode, with the following ionic transitions: acrylamide (*m*/*z* 72.01 > *m*/*z* 54.93); ^13^C_3_-acrylamide (*m*/*z* 75.01 > *m*/*z* 57.89) and HMF (*m*/*z* 126.98 > *m*/*z* 108.77). All analyses were performed in duplicate and the results were expressed as ng/g sample.

#### 3.3.3. Analytical Method Validation

The validation of the method included the following parameters: apparent recovery (R_A_), extraction efficiency (R_E_), matrix effect (SSE) (signal suppression/enhancement), intra- and inter-day precision (repeatability and reproducibility), as well as limit of detection (LOD) and limit of quantification (LOQ) and linearity (working range) [42,43]. The recovery experiments were performed by analyzing spiked samples and extracts with two different concentrations (5 and 10 ng/g for acrylamide and ^13^C_3_-acrylamide; 100 and 250 ng/g for HMF) in six replications.

The performance characteristics R_A_, R_E_ and SSE were calculated according to the following equations [43,44]:

(1) $$R_{A}=\frac{{\mathrm{area}}_{\mathrm{spiked}\;\mathrm{sample}}}{{\mathrm{area}}_{\mathrm{standard}}}$$

(2) $$R_{E}=\frac{{\mathrm{area}}_{\mathrm{spiked}\;\mathrm{sample}}}{{\mathrm{area}}_{\mathrm{spiked}\;\mathrm{extract}}}$$

(3) $$\mathrm{SSE}=\frac{{\mathrm{area}}_{\mathrm{spiked}\;\mathrm{extract}}}{{\mathrm{area}}_{\mathrm{standard}}}$$

The intra-day precision was expressed as the relative standard deviation (RSD) calculated from six replicates of the spiked samples, while the inter-day precision was calculated as RSD between two different days of analysis [42,45,46].

Standard solutions of acrylamide, ^13^C_3_-acrylamide and HMF were used to construct the calibration curves. LOD (Limit of detection) and LOQ (Limit of quantification) values were estimated from the regression equations, which were calculated from the calibration curves according to the equations [45,47]:

(4) $$\mathrm{LOD}=\frac{3.3\sigma }{S}$$

where: σ = the standard deviation of the response, S = the slope of the calibration curve.

(5) $$\mathrm{LOQ}=\frac{10\sigma }{S}$$

where: σ = the standard deviation of the response, S = the slope of the calibration curve.

### 3.4. Statistical Analysis

Experimental data were analyzed using one-way analysis of variance (ANOVA) and Fisher’s least significant difference (LSD), with the significant difference of the data at *p* < 0.05 in the software program STATISTICA 13.3 (StatSoft, Inc., Tulsa, OK, USA) and Microsoft Office Excel 2016.

## 4. Conclusions

This work represents the first published LC-MS/MS method for the simultaneous determination of acrylamide and HMF in one step. According to the validation results obtained for the apparent recovery (R_A_), extraction efficiency (R_E_) and matrix effect (SSE), for which mean percentages exceeded 90% for both analytes, as well as satisfactory values for intra- and inter-day precision, it can be concluded that the developed method is suitable for the detection of relevant concentrations of acrylamide and HMF. In this study, the method is applied for the determination of acrylamide and HMF in non-extruded and extruded products, showing that the extrusion process caused the formation of these analytes. However, their formation depends on the raw materials used, so it is evident that the application of food industry by-products—brewer’s spent grain (BSG), sugar beet pulp (SBP) and apple pomace (AP)—in the production of corn snack products resulted in higher contents of both compounds. The obtained results represent a significant scientific contribution, since to date no research has been published on acrylamide and HMF in extrudates with the addition of the investigated by-products. Although there are no maximum permissible contents of acrylamide and HMF for corn snack products, the values obtained in this study were significantly lower than the values prescribed for other related product groups. Accordingly, these results indicate that the extrusion process can be used for the production of corn snack products enriched with the by-products which are safe for the consumer, with low contents of these potentially harmful compounds for the human organism.

## Figures and Tables

**Table 1:** Validation parameters of the used LC-MS/MS method.

Validation Parameters *		Acrylamide	^13^C_3_-acrylamide	HMF
Calibration curve equation		y = 6421.6x − 2739.3	y = 7114.5x − 2567.8	y = 205.38x + 6249.7
R^2^		0.997	0.998	0.999
LOD (ng/g)		0.62	0.58	18.9
LOQ (ng/g)		1.89	1.75	57.5
Retention time (min)		9.10 ± 0.07	9.10 ± 0.06	13.3 ± 0.08
R_A_ (%)	Lower concentration	91.2	91.9	90.0
	Higher concentration	91.6	92.3	90.9
	Average	91.4	92.1	90.4
R_E_ (%)	Lower concentration	92.7	93.1	91.2
	Higher concentration	93.0	93.9	94.2
	Average	92.8	93.5	92.7
SSE (%)	Lower concentration	98.5	98.8	98.7
	Higher concentration	98.5	98.3	96.5
	Average	98.5	98.5	97.6
RSD Intraday (%)	Lower concentration	2.89	2.77	3.17
	Higher concentration	2.86	2.91	3.15
	Average	2.88	2.84	3.16
RSD Interday (%)	Lower concentration	3.12	3.01	3.36
	Higher concentration	3.06	3.08	3.43
	Average	3.09	3.05	3.40

R^2^—regression coefficient; LOD—limit of detection; LOQ—limit of quantification; R_A_—apparent recovery; R_E_—extraction efficiency; SSE—signal suppression/enhancement; RSD—relative standard deviation; * *n* = 6 for recovery and precison experiments at two concetration levels (lower concentration: 5 ng/g for acrylamide and ^13^C_3_-acrylamide and 100 ng/g for HMF; higher concentration: 10 ng/g for acrylamide and ^13^C_3_-acrylamide and 250 ng/g for HMF.

**Table 2:** Acrylamide and HMF contents in raw materials.

Sample *	Acrylamide (ng/g)	HMF (ng/g)
Corn grits	<LOD	63.0 ± 3.00 ^a^
BSG	<LOD	5634 ± 11.0 ^a^
SBP	<LOD	658 ± 15.8 ^a^
AP	<LOD	16,019 ± 978 ^b^

<LOD—below limit of detection; BSG—brewer’s spent grain, SBP—sugar beet pulp, AP—apple pomace; * Values with different letters in the same column are significantly different at *p* < 0.05.

**Table 3:** Acrylamide and HMF contents in non-extruded and extruded samples.

Sample*	Non-Extruded		Extruded	
	Acrylamide (ng/g)	HMF (ng/g)	Acrylamide (ng/g)	HMF (ng/g)
Corn grits	<LOD	63.0 ± 3.00 ^a^	2.25 ± 0.29 ^a^	174 ± 2.21 ^a^
Corn + 5% BSG	<LOD	68.2 ± 3.31 ^a^	2.74 ± 0.02 ^a,b^	192 ± 5.36 ^a^
Corn + 10% BSG	<LOD	78.4 ± 4.89 ^a^	2.86 ± 0.05 ^a,b^	290 ± 18.9 ^a^
Corn + 15% BSG	<LOD	83.8 ± 4.89 ^a^	3.13 ± 0.23 ^b^	301 ± 12.6 ^a^
Corn + 5% SBP	<LOD	86.0 ± 0.47 ^a^	2.59 ± 0.02 ^a,b^	173 ± 11.2 ^a^
Corn + 10% SBP	<LOD	88.1 ± 1.26 ^a^	2.68 ± 0.07 ^a,b^	224 ± 30.5 ^a^
Corn + 15% SBP	<LOD	90.6 ± 0.32 ^a^	3.00 ± 0.21 ^b^	317 ± 9.47 ^a^
Corn + 5% AP	<LOD	421 ± 20.5 ^b^	3.98 ± 0.07 ^c^	971 ± 85.2 ^b^
Corn + 10% AP	<LOD	801 ± 37.9 ^c^	4.93 ± 0.71 ^d^	2622 ± 110 ^c^
Corn + 15% AP	<LOD	1277 ± 11.0 ^d^	5.37 ± 0.50 ^d^	6069 ± 789 ^d^

<LO—below limit of detection; BSG—brewer’s spent grain, SBP—sugar beet pulp, AP—apple pomace; * Values with different letters in the same column are significantly different at *p* < 0.05.
